# A novel medium size lactam ring analoges as antibacterial agents: Synthesis, biological evaluation and molecular docking studies

**DOI:** 10.17179/excli2017-193

**Published:** 2017-08-14

**Authors:** Putcha A. N. V. Harita, Putcha Seshi Kumar, Shiva Krishna Reddy Guduru, Parameshwar Ravula, J N Narendra Sharath Chandra

**Affiliations:** 1 Department of Medicinal Chemistry, Guru Nanak Institutions Technical Campus, School of Pharmacy, Jawaharlal Nehru Technological University, Hyderabad-501301, India; 2 Shantha Biotechnics private limited. A Sanofi company, Medchal, India-501401; 3 Dr. Reddy's Institute of Life Sciences, University of Hyderabad Campus, Gachibowli, Hyderabad, India-500 046; 4 Department of Pharmaceutical Chemistry, Bharat Institute of Technology, Mangalpally, Telanagana, India-501510

**Keywords:** medium size ring, synthesis, coupling reaction, lactam, bacillus subtilis, anti-bacterial, docking

## Abstract

A novel series of medium size (S)-3-alkyl-3,4,6,7-tetrahydro-1H-benzo[e][1,4]diazonine-2,5-dione **(6a-f)** analogues were synthesized from (E)-3-(2-nitrophenyl)acrylicacid **(2)** reacting with various amino acid esters using Di-isopropyl Carbodiimide as a coupling agent. The final cyclization is carried out by using reagent 1-Ethyl-3(3-dimethylaminopropyl) Carbodiimide Hydrochloride. The synthesized compounds have been supported by Mass, ^1^H-NMR and^ 13^C-NMR. Further antibacterial studies were conducted, where some molecules are noticed with potent activity, especially molecule **6d** shown highest activity which was also supported by molecular docking studies. All final molecules were docked with enzyme peptide deformylase to determine the probable binding conformation.

## Introduction

Over the years, antibacterial agents are playing a vital role to treat infectious diseases (Woon and Fisher, 2016[23]). Apart from the increasing new diseases, emerging resistance in bacteria against available antibacterial agents is a world-wide problem (Lin et al., 2015[8]; Brickner et al., 1996[1]; Neu, 1985[13]). Even though the design, development and expansion of a number of new antibacterial agents against such bacteria, their clinical value is inadequate to treat an exceeding group of life threatening systemic infections because of their comparatively high risk of toxicity (Sanders and Sanders, 1979[20]), emergence of drug resistant strains (Ventola, 2015[22]; Neu, 1992[14]), pharmacokinetic differences, and insufficiencies in their activity (Bush, 2004[2]; Levison and Levison, 2009[7]). Our research community is trying to synthesize molecules which are having better pharmacokinetic properties and are sufficient in their activity.

**Figure 1:**
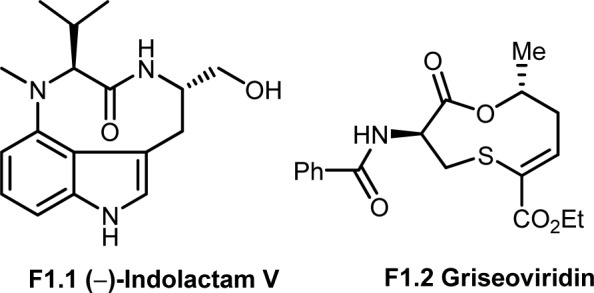
9-membered ring containing molecules in nature

Literature results show, the 9-membered medium size ring molecules and macrocycles are bioactive and have promising therapeutic potential value (Driggers et al., 2008[3]). Especially macrocycles provide pre-organized ring structure to molecules and are ideal for binding protein surface (Reayi and Arya, 2005[19]; Yu and Sun, 2013[24]; Marsault and Peterson, 2011[10]; Kopp et al., 2012[6]). Meseguer and co-workers (1999[12], 2000[11]), synthesized libraries of indolactam analogs, where the 9-membered ring structure is present in a natural product Indolactam V (**F1.1**), the core structure of tumor-protecting teleocidins. Marcantoni and co-workers (2000[9]) synthesized a 9-membered medium size macrocyclic ring component of Griseoviridin (**F1.2**) which is a broad spectrum antibiotic with *in vitro* inhibitory activity toward various pathogenic fungi and bacteria (Figure 1(Fig. 1)). 

**Figure 2:**
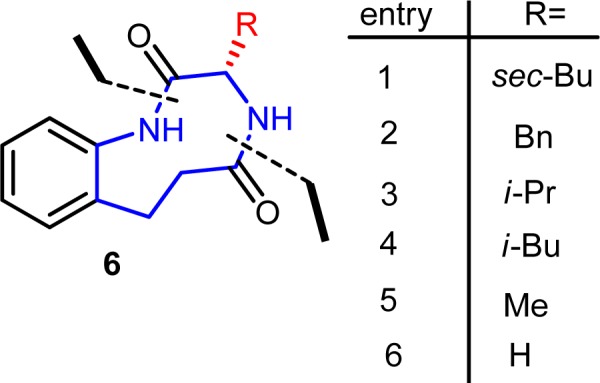
Retrosynthesis of 9-membered ring molecule

A series of medium size ring [(S)-3-alkyl-3,4,6,7-tetrahydro-1H-benzo[e][1,4]diazonine-2,5-dione] molecules (**6a-f**) was synthesized on benzene ring and screened for antibacterial activity. The key reactions that are involved for constructing this (medium size) ring are reduction of nitro group and amino ester coupling reactions (Figure 2(Fig. 2)).

(S)-3-alkyl-3,4,6,7-tetrahydro-1H-benzo [e] [1,4]diazonine-2,5-dione analogues (**6a-f**) are synthesized from intermediate (E)-3-(2-nitrophenyl)acrylic acid (**2**) reacting with various amino acid ester. The intermediate (E)-3-(2-nitrophenyl)acrylic acid (**2**) is in-turn synthesized from *o*-Nitrobenzaldehyde. The final step cyclization is carried out using reagent 1-ethyl-3(3-dimethylaminopropyl) carbodiimide hydrochloride and acetonitrile as solvent. Further, the synthesized compounds were screened for antibacterial activity. 

## Materials and Methods

### Chemistry

All solvents and reagents were used, as received from the suppliers and some of them were distilled before use. All the reactions were monitored by thin layer chromatography (TLC) on pre-coated silica gel 60F254 (Merck, 1.05554) and the spots were visualized with UV light at 254 nm or alternatively by staining with ninhydrine or potassium permanganate or ceric ammonium molybdate. Flash column chromatography was performed using silica gel (Merck, 60A, 230-400 Mesh). All reactions were performed in oven dried glassware. Dimethylformamide, dichloromethane, methanol and tetrahydrofuran were dried immediately prior to use according to standard procedures: Dimethylformamide, dichloromethane was distilled under N_2_ from CaH_2_, methanol was distilled under N_2_ over Mg and tetrahydrofuran was distilled under N_2_ over Na. All solvents were removed by evaporation under reduced pressure. ^1^H-NMR and ^13^C-NMR spectra were recorded on Varian 400 MHz CDCl_3_ using TMS as internal standard. Electron impact (EI) and chemical ionization mass spectra were recorded on an Agilent-6430 mass spectrometer instrument.

#### General procedure for synthesis of (E)-ethyl 3-(2-nitrophenyl)acrylate (1)

To a solution of sodium hydride in dry tetrahydrofuran and triethyl phosphonoacetate was added at 0 °C, and stirred under nitrogen atmosphere for 30 min. A solution of 2-nitro-benzaldehyde in tetrahydrofuran was then added drop wise to the reaction mixture and allowed to stir for 4 h at 0 °C. After completion of the reaction (monitored through TLC), the reaction mixture was quenched by adding saturated ammonium chloride solution and it was extracted with ethyl acetate. The collected organic layers were washed with brine solution, and then dried over anhydrous sodium sulphate. The solution was filtered and concentrated, which was then purified by column chromatography to give a yellow liquid compound.

#### General procedure for synthesis of (E)-3-(2-nitrophenyl)acrylic acid (2)

To the solution of ester in methanol and water, lithium hydroxide monohydrate was added. The reaction mixture was stirred at 50 °C for 12 h. After completion of reaction (monitored through TLC), methanol was removed under reduced pressure. The obtained aqueous solution was added with HCl till the pH is in between 5-6 and then extracted with ethyl acetate. The combined organic phase were dried over sodium sulfate_, _filtered and concentrated to obtained light yellow colour solid compound.

#### General procedure for synthesis of compounds (3a-f)

To a suspension of compound **(2) **in dry dimethylformamide, amino ester, diisopropyl carbodiimide, 1-hydroxy-1H-benzotriazole and N, N-diisopropylethylamine were added at 0 °C and allowed to stirred for 12 h under nitrogen atmosphere. After completion of the reaction (which was monitored through TLC), solution was quenched with sodium bicarbonate solution and further extracted with ethyl acetate. The obtained organic layer was washed with brine solution, and then dried over anhydrous sodium sulphate. The solution was filtered and concentrated to leave a crude oil. The obtained liquid was purified by column chromatography to give the pure compound.

#### General procedure for synthesis of compounds (4a-f)

To a suspension of compound **(3a-f)** in absolute ethanol, 10 % Pd/C was added and stirred the reaction mixture for 12 h under hydrogen atmosphere at room temperature. After completion of the reaction (monitored through TLC), the reaction mixture was passed through celite and concentrated. The crude compound was subjected to column chromatography to give white colour oily compound.

#### General procedure for synthesis of compounds (5a-f)

To the suspension of ester **(4a-f)** in Tetrahydrofuran and water, Lithium hydroxide Monohydrate was added. For 12 h the reaction mixture was stirred at room temperature. The reaction was monitored through TLC. Later after completion of reaction, the solution was filtered and extracted with ethyl acetate. The obtained liquid was dried over sodium sulfate, filtered and concentrated.

#### General procedure for synthesis of compounds (6a-f)

To the suspension of acid **(5a-f)** in acetonitrile, 1-ethyl-3(3-dimethylaminopropyl) carbodiimide hydrochloride were added at room temperature and allow to stir under nitrogen atmosphere for 12 h. After finishing of reaction (which was monitored through TLC), the solution was quenched with sodium bicarbonate solution. It was then concentrated and extracted with ethyl acetate. The collected organic layer was washed with brine solution and it was dried over anhydrous sodium sulphate. The solution was then filtered and concentrated to give a crude oil, later purified by column chromatography to give pure compound.

### (S)-3-isobutyl-3,4,6,7-tetrahydro-1H-benzo[e][1,4]diazonine-2,5-dione (6a)

Molecular Formula : C_15_H_20_N_2_O_2_;R*f*: 0.2 at 0.2: 9.8 ethyl acetate/DCM; Yield : 26 %;^ 1^H-NMR (400 MHz, CDCl_3_) δppm : 8.24 (d, *J = *8.01 Hz, 1H), 7.38-7.09 (m, 4H), 4.20 (m, 1H), 3.02-2.95 (m, 2H), 2.88 (m, 2H), 2.05 (m, 1H), 1.82 (m, 1H), 1.54 (m, 1H), 1.02 (m, *J = *17.83, 6.64 Hz, 6H); ^13^C-NMR (100 MHz, CDCl_3_) δppm : 180.6, 160.6, 133.7, 128.3, 127.8, 126.2, 125.2, 117.5, 67.2, 40.7, 29.7, 25.9, 25.2, 23.2, 21.7; LRMS : (ES+) m/z = 242.8 (Fragmented ion).

### (S)-3-benzyl-3,4,6,7-tetrahydro-1H-benzo[e][1,4]diazonine-2,5-dione (6b)

Molecular Formula : C_18_H_18_N_2_O_2_; R*f*: 0.2 at 3 : 7 of ethyl acetate/hexanes; Yield : 10 %; ^1^H-NMR (400 MHz,CDCl_3_) δppm : 8.16 (d,* J = *8.15 Hz, 1H),7.32-7.07 (m, 9H), 4.50-4.44 (s, 1H),3.36 (m, 1H), 3.13 (m, 1H), 2.77 (m,4H); ^13^C-NMR (100 MHz,CDCl_3_) δppm : 179.2, 161.2, 136.0, 133.4, 129.5, 128.2, 128.1, 127.7, 126.7, 126.2, 125.3, 117.5, 69.2, 37.2, 29.7, 25.7, 25.7; LRMS : (ES+) m/z = 276.7 (Fragmented ion).

### (S)-3-isopropyl-3,4,6,7-tetrahydro-1H-benzo[e][1,4]diazonine-2,5-dione (6c)

Molecular Formula : C_14_H_18_N_2_O_2_; R*f*: 0.3 at 3 : 7 ethyl acetate/hexanes; Yield : 46 %; ^1^H-NMR (400 MHz, CDCl_3_) δppm : 8.25 (d, *J = *8.13 Hz, 1H), 7.37-7.28 (m, 2H), 7.24 (d, *J = *7.31 Hz, 1H), 7.16 (t, *J = *7.46 Hz, 1H), 4.10 (m, 1H) , 2.98 (m, *J = *7.47 Hz, 2H), 2.95-2.87 (m, 2H), 2.45-2.37 (m, 1H), 0.90 (m, 6H); ^13^C-NMR (100 MHz, CDCl_3_) δppm : 179.5,161.2, 133.7, 128.3, 127.8, 126.2, 125.2, 117.5, 73.6, 29.7, 26.1, 19.3, 16.8, 14.1; LRMS : (ES+) m/z = 228.7 (Fragmented ion).

### (S)-3-sec-butyl-3,4,6,7-tetrahydro-1H-benzo[e][1,4]diazonine-2,5-dione (6d)

Molecular Formula : C_15_H_20_N_2_O_2_; R*f *: 0.5 at 3 : 7 ethyl acetate/hexanes; Yield : 10 %;^1^H-NMR (400 MHz, CDCl_3_) δppm : 8.26 (m, 1H), 7.29 (m, 3H), 7.15 (t, *J = *7.47 Hz, 1H), 4.26-4.13 (d, 1H), 3.02-2.87 (m, 5H), 2.17 (m, 1H), 1.70 (m, 1H), 1.53-1.43 (m, 1H), 1.10 (d, *J = *6.90 Hz, 1H), 1.04 (t, *J = *7.43 Hz, 2H), 0.95 (t, *J = *7.39 Hz, 1H), 0.81 (d, *J = *6.81 Hz, 2H); ^13^C-NMR (400 MHz, CDCl_3_) δppm : 180.1, 161.1, 133.8, 128.2, 127.7, 126.1, 125.2, 117.5, 71.9, 37.2, 26.6, 25.9, 24.5, 13.9, 11.9; LRMS : (ES+) m/z = 243.3 (Fragmented ion).

### (S)-3-methyl-3,4,6,7-tetrahydro-1H-benzo[e][1,4]diazonine-2,5-dione (6e)

Molecular Formula : C_12_H_14_N_2_O_2_; R*f*: 0.2 at 3 : 7 ethyl acetate/hexanes; Yield : 40 %; ^1^H-NMR (400 MHz, CDCl_3_) δppm : 8.23 (d, *J = *8.13 Hz, 1H), 7.39-7.30 (m, 2H), 7.20 (d, *J = *7.31 Hz, 1H), 7.15 (t, *J = *7.46 Hz, 1H), 4.17 (m, 1H) , 2.95 (m, *J = *7.47 Hz, 2H), 2.90-2.85 (m, 2H),1.34(d, 3H); ^13^C-NMR (100 MHz, CDCl_3_) δppm : 178.5,161.2, 134.7, 128.3, 127.8, 125.2, 123.8, 115.5, 72.6, 30.7, 25.1, 18.8; LRMS : (ES+) m/z = 200.5 (Fragmented ion).

### 3,4,6,7-tetrahydro-1H-benzo[e][1,4]diazonine-2,5-dione (6f)

Molecular Formula : C_11_H_12_N_2_O_2_; R*f*: 0.35 at 3 : 7 ethyl acetate/hexanes; Yield : 54 %; ^1^H-NMR (400 MHz, CDCl_3_) δppm : 8.20 (d, *J = *8.12 Hz, 1H), 7.38-7.31 (m, 2H), 7.25 (d, *J = *7.31 Hz, 1H), 7.14 (t, *J = *7.55 Hz, 1H), 4.15 (d, 2H) , 2.93 (m, *J = *7.47 Hz, 2H), 2.91-2.85 (m, 2H); ^13^C-NMR (100 MHz, CDCl_3_) δppm : 180.5,165.2, 136.8, 129.1, 127.8, 124.7, 122.8, 112.5, 74.6, 38.7, 25.7; LRMS : (ES+) m/z = 185.7 (Fragmented ion).

### Antibacterial activity

The title compounds were subjected for antibacterial activity, where Zone of inhibition (Pandey et al., 2010[15]; Kirby and Bauer, 1996[5]) and Minimum inhibitory concentration (Sarker et al., 2007[21]; Gibbons et al., 2002[4]; Prachayasittikul et al., 2010[16]) test were conducted against test organism *Bacillus subtilis* which is a gram positive bacteria, by using Cup Plate Method and Microtitre Plate Method respectively. 

### Determination of Zone of Inhibition - Cup Plate Method

Cultures were sub-cultured on Nutrient Agar (NA) and Sabouraud Dextrose Agar (SDA) plates and further stored in slants as stock cultures. For the experiment, stock cultures were prepared by inoculating each culture from slants to flask in sterile Nutrient Broth (NB) and incubated at 37 °C for 24 h (Bacteria). The stock cultures were adjusted to 0.3 OD (Bacteria) at 650 nm by using spectrophotometer and used for assay. Sterile NA plates where prepared at 0.1 ml of inoculums from standardized culture of test organism and were spread evenly. A sterile borer was used to prepare wells with diameter 10 mm and 100 µl (in order to get the concentration of 1000, 500, 250 µg/well) of the test material, standard antibiotic. A standard antibiotic, i.e., ciprofloxacin was tested against bacteria and was added in each well separately. At 4 °C the plates were kept for 1 h to carry out the diffusion of test solution into the medium, so that the plates were incubated at a temperature best for the growth of test organism for a period of time enough for the growth of at least 10 to 15 generations. The zone of inhibition of microbial growth around the well was measured in mm.

### Determination of Minimum Inhibitory Concentration (MIC) - Microtitre Plate Method

A sterile 96 well plate, prepared under aseptic conditions, was labelled. Into the first row of the plate a quantity of 100 µL of test material in DMSO (generally a stock concentration of 0.2 mg/ml for purified material and 2 mg/mL for crude extracts) was pipetted. 50 µL of sterile broth was added to all other wells. Sterile dilutions were performed using multichannel pipette. Discard the tips after use, so that each well had 50 µL of the test compound in consecutively descending concentrations. Resazurin indicator solution was added to each well of 10 µL. A 30 µL of sterile broth was added using a pipette. Lastly, microbial suspension of 10 µL was added to each well. By using cling film each plate was wrapped loosely to protect the culture from dehydration. Each plate has a set of positive, negative and standard solution. The prepared plates were placed in an incubator at temperature 37 °C for 18-24 h. The change in colour was then observed visually. The colour changes from purple to pink or colourless is taken as positive. The lowest concentration at which change in colour occurred was considered as MIC value.

### Molecular docking

Docking studies were carried on windows 2002 with MOE 2008.10 version according to reported method (Ravula et al., 2016[17][18]). From the protein data bank (PDB code: 1G27), peptide deformylase enzyme was taken and visualized the receptor by using sequence option and deleted further co-factors. The ligand structures were written by using a builder module and the partial charges were adjusted and subsequently according to the standard geometry, 3D protonation and hydrogen addition was performed. At cut off 12 using force field MMFF94x at 0.01KJ mole gradient the ligand's energy minimized. By using the option simulation docking was performed and was followed by using sequence option dock on selected active site amino acids were performed and eventually docked using setting options such as alpha triangle, receptor and solvent, affinity dG, selected residues, force field refinement and best 30 pose. After achieving docking results, best pose was retained out of the 30 best posed results for each chemical structure. The resultant best pose scores wereused for further examinination of docking and interaction.

## Results and Discussion

**Figure 3:**
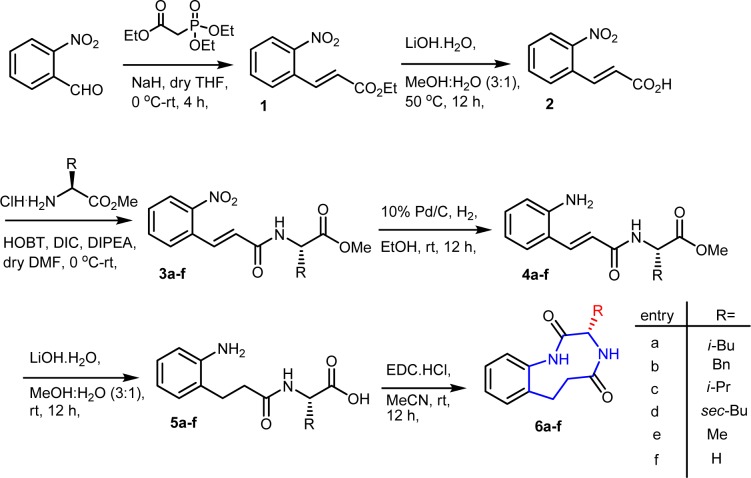
Figure 3: Synthesis of (S)-3-alkyl -3,4,6,7-tetrahydro-1H-benzo[e] [1,4]diazonine-2, 5-dione analogues (6a-f)

Synthesis started from the commercially available *o*-Nitrobenzaldehyde, it is subjected to Horner-Wittig reaction followed by ester hydrolysis obtained (E)-3-(2-nitrophenyl)acrylic acid (**2**). This was coupled with various amino acid esters with the help of 1-hydroxy-1H-benzotriazole and di-isopropyl carbodiimide coupling reagents obtained **(3a-f)** in a good yield. This coupled product subjected to hydrogenation on Pd/C which results in one step nitro group and double bond reduction. The resulted ester **(4a-f)** again hydrolysed with Lithium hydroxide condition obtained acid intermediate **(5a-f)**. Finally ring closed with help of 1-Ethyl-3(3-dimethylaminopropyl) carbodiimide hydrochloride coupling reagent obtained a medium size ring (**6a-f)** on benzene (Figure 3(Fig. 3)). The final synthesized molecules were purified by column chromatography and were analyzed by physical and analytical methods.

### Biological evaluation

**Figure 4:**
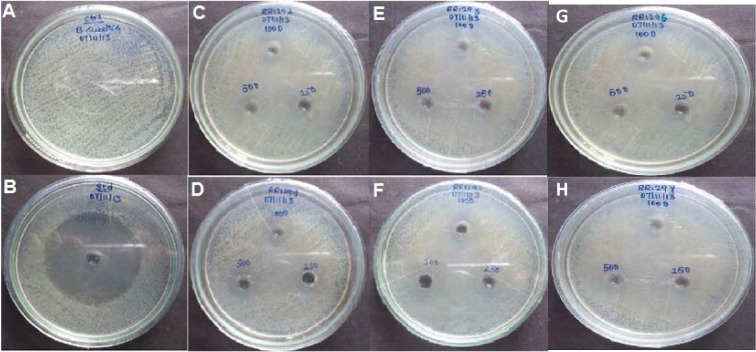
Zone of inhibition assay against *B. subtilis *by cup plate method

**Figure 5:**
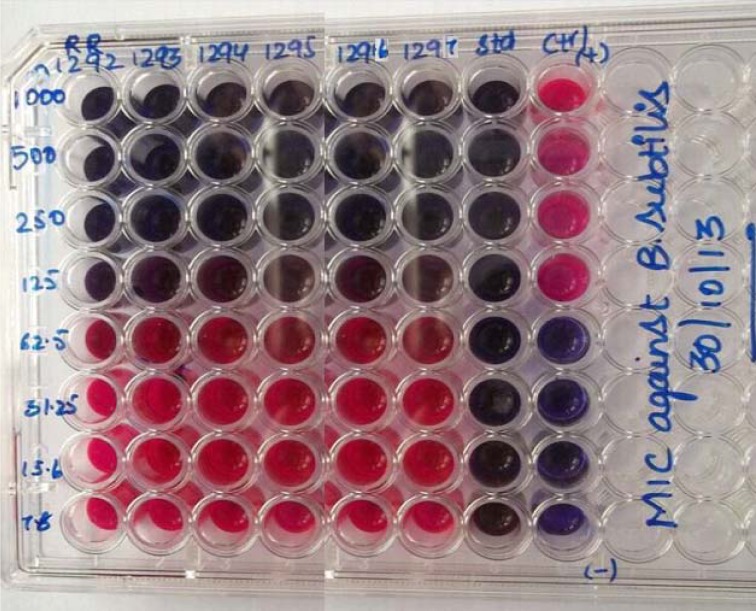
Figure 5: Minimum Inhibitory Concentration activity of test samples against *B. subtilis* at different concentrations

The newly synthesized compounds **6a-f** were evaluated for their antibacterial activity (zone of inhibition and Minimum Inhibitory Concentration) against *Bacillus subtilis*, a gram positive organism. The screening data have been shown in Table 1(Tab. 1), indicated that the compound **6d **showed significant antibacterial activity. Remaining compounds were found to possess moderate activity. Ciprofloxacin was used as standard, for both the test, its Minimum Inhibitory Concentration value is >7.8 µg/ml, whereas the title molecules are observed with value is 125 µg/ml shown in Figure 4(Fig. 4) and 5(Fig. 5).

### Molecular docking

**Figure 6:**
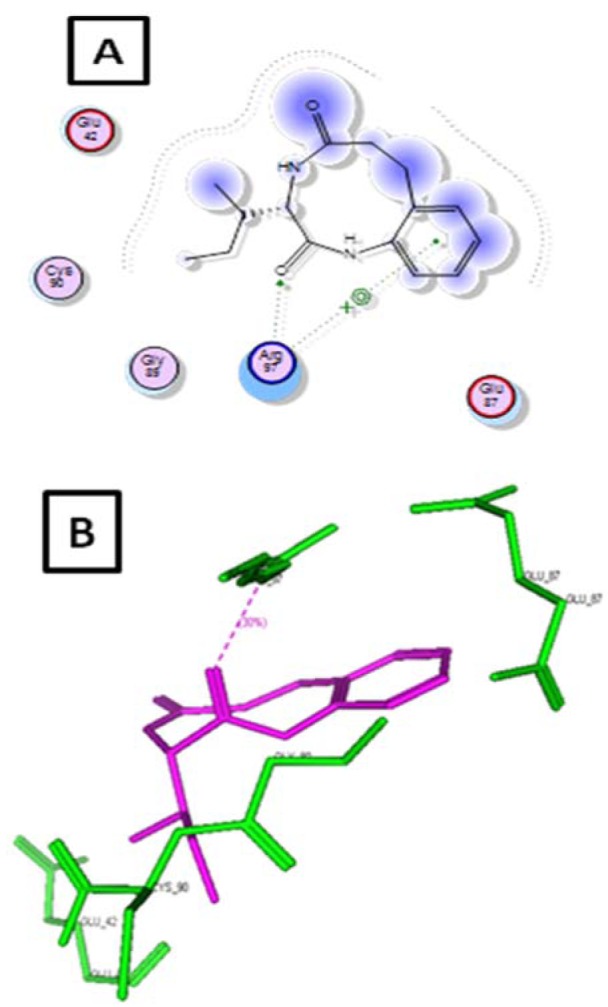
(A) Two-dimentional representation of the interaction mode of 6d with peptide deformylase enzyme. (B) Three-dimentional structural model of compound 6d (purple) into peptide deformylase enzyme.

Molecular docking study was carried for further study of action of final compounds with peptide deformylase enzyme and elucidated the observed biological results. Comparing of all final molecules, the docking results of compound **6d** showed better interactions of one hydrogen bond interaction i.e., oxygen of C=O and Arg 97; d=2.47A°. Benzene ring showed stacking interaction with Arg 97. Moreover molecule was surrounded by Glu 42, Cys90, Gly89 and Glu 87. This overall supports the results obtained for antibacterial activity (Figure 6(Fig. 6)).

## Conclusion

The novel analogues of lactam containing medium size ring molecules were synthesized and screened for antibacterial activity. Of all final compounds, compound **6d** showed good bacterial inhibition and other compounds showed moderate inhibition. This was supported by molecular docking studies. With further molecular modification and QSAR of these compounds, several other promising bioactive molecules can be developed in future.

## Figures and Tables

**Table 1:**
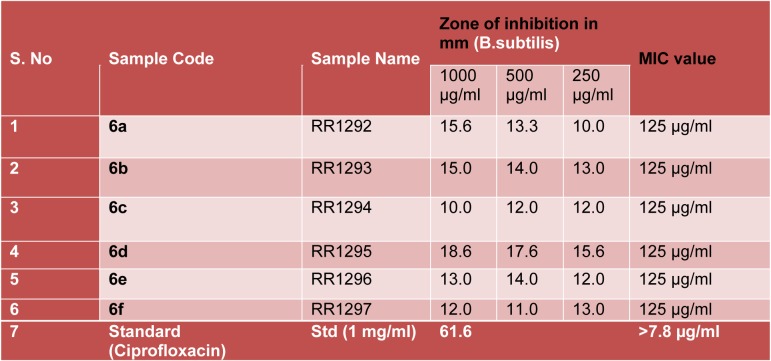
Zone of inhibition and MIC value of final cyclized molecules
